# Prediction of Spatiotemporal Changes in Sloping Cropland in the Middle Reaches of the Yangtze River Region under Different Scenarios

**DOI:** 10.3390/ijerph20010182

**Published:** 2022-12-23

**Authors:** Xiaowei Yao, Ting Luo, Yingjun Xu, Wanxu Chen, Jie Zeng

**Affiliations:** 1 School of Public Administration and Laws, China University of Geosciences (Wuhan), Wuhan 430074, China; 2 Key Laboratory of Legal Research of the Ministry of Natural Resources, Wuhan 430074, China; 3 School of Geography and Information Engineering, China University of Geosciences (Wuhan), Wuhan 430074, China; 4 State Key Laboratory of Earth Surface Processes and Resource Ecology, Beijing Normal University, Beijing 100875, China

**Keywords:** sloping cropland, slope spectrum, SSP-RCP, PLUS, driving factors, middle reaches of the Yangtze River region (MRYRR)

## Abstract

With the rapid urban expansion and extensive occupation of cropland, sloping cropland has become an important cropland resource across China. How sloping cropland will change under different socioeconomic scenarios is poorly understood. Therefore, we modeled land-cover change using SSP-RCP multi-scenario simulations and analyzed the evolution and driving factors of sloping cropland change in the middle reaches of the Yangtze River Region (MRYRR). The results indicate the following: In the past twenty years, the cropland and sloping cropland areas in this region declined but the proportion of sloping cropland in total area has been increasing. The average slope of sloping cropland has increased from 7.95° to 8.28°. By 2035, the sloping cropland and total cropland areas will continue to decrease according to the current trend (SSP2-4.5). The average slope will increase maximally to 8.63° under the SSP4-3.4 scenario and minimally to 8.45° under the SSP4-6.0 scenario. Under SSP4-3.4, the extent of slope increase will exceed that in 2005–2010, when regional cropland slope showed the strongest increase in the past. Among 14 social, economic, and ecological factors, average annual precipitation and GDP contributed the most to the change in sloping cropland. This study provides support for decision-making in sustainable land resource allocation to balance urban expansion and cropland conservation.

## 1. Introduction

With the rapid process of industrialization and urbanization in the last four decades in China, there have been great achievements in social and economic development [1]. While agriculture has also achieved persistent bumper harvests [2,3,4], the distribution of cropland resources has changed dramatically throughout the country [5,6]. As the accelerated urbanization has encroached on the cropland in the countryside surrounding cities and towns via construction, large amounts of farmland have been transferred from the south to the north [7], and from urbanizing areas, mostly in plain regions with high soil quality, to the remote rural hinterland with relatively low soil quality [8], that are unlikely to be occupied again by urban expansion and human activities. This continuous land-use transformation is associated with an increasing amount of sloping cropland [9], which is defined as cropland with a slope greater than 2° [10]. Studies have shown that sloping cropland has become a crucial resource for grain production [10,11,12] and maintaining people’s basic livelihoods [13,14]. It is widely recognized that an increase in the slope gradient results in more surface runoff and soil loss [12,15], causing lower soil fertility and a higher risk of eutrophication in rivers, lakes, and coastal areas [2]. Soil loss especially from sloping cropland is considered to be the main source of river sediment [15], so the Chinese central government has made great efforts to control water erosion and reduce cultivation in sloping areas, such as the Sloping Land Conversion Program, which is believed to be the world’s largest payment for ecosystem services program for improving ecological conditions and farmers’ livelihood [16,17]. Even though farmers are willing to give up cultivation of sloping land by diversifying their livelihood options [18], sloping croplands are still commonly found in central and southern China due to the bottom line of grain security and local terrain restrictions. In the meantime, local governments have aimed to expand cultivation to new fertilized croplands in rural areas and improve their quality through engineering approaches, such as the Project of Transforming Slope Into Terrace. The areas, quality, and spatial distribution of sloping cropland greatly impact soil and water conservation [19], along with regional food security [20]. However, the spatial distribution of sloping cropland is unstable due to complex natural and social factors, and the sustainable utilization of sloping cropland is sensitive to disturbance by human activities [21]. Consequently, the evolution characteristics and driving factors of sloping cropland distribution in the past must be thoroughly studied and future trends under different climate-change and developmental scenarios must be predicted.

Numerous studies have analyzed the process of cropland changes [22,23,24] and revealed the impacts of different urbanization stages on cropland losses at various spatial scales [25,26]. Some characterized the spatiotemporal variation and explored the optimization of cropland patterns, which is the premise of sustainable societal development [27]. In recent years, several studies have focused on mapping cropping intensity [28], quality patterns of cropland [29] by integrating multiple data sources, and cropland use efficiency while minimizing the associated environmental impacts [4,30]. Regarding the distribution of sloping cropland, studies have focused more on the evaluation of the cultivated layer’s soil quality [31], soil and water dynamics [32,33] and soil loss in sloping farmland [12,34,35], ignoring the characteristics of farmland on a vertical-gradient terrain. Only a few studies have shown the spatiotemporal changes in sloping cropland in some provinces of China [10,20], but they simply described the variations in the amounts of cropland at different slopes in the past without predicting them under various development situations. As a result, there is still a paucity of thorough studies on the spatial characteristics of sloping cropland, both in the past and future, especially at relatively large regional scales.

The choice of models for land-use simulations, particularly for urban–rural land conversion, has been a hot topic in international research. Cellular automata (CA) have been used extensively to predict the spatial and temporal changes in land use with a group of natural and social economic factors [36,37]. However, they are more applicable for simulating urban land expansion and transformation within urban land-use types [38]. More importantly, our understanding of the underlying causes of land-use changes by CA remains still inadequate, even though many studies have focused on improving model accuracy [39,40]. The latest Patch-generating Land-use Simulation (PLUS) model with the original features of the CA model can better capture the driving mechanisms of land-use change by integrating a rule-mining framework called the Land Expansion Analysis Strategy (LEAS), and is thus able to improve the precision of simulation. Another advantage of the PLUS model is its ability to emulate patch-level changes in land use by adding a new patch seeding mechanism called multi-type Random Seeds (CARS) [41]. Therefore, this newly developed model has been widely used in urban and rural land use projection [42,43].

In order to conduct more accurate analysis and gain a better understanding of the changing trends of sloping cropland in the future based on different contexts, we applied the scenario projections of sloping cropland change under the combined framework of the shared socioeconomic pathways and representative concentration pathways (SSP-RCP) as the scenario combinations, which provide different or even contrasting socioeconomic and climatic conditions [44,45]. They are included in the latest World Climate Research Program Coupled Model Intercomparison Project Phase 6 (CMIP6) released by the Intergovernmental Panel on Climate Change (IPCC) in 2020, comprising quantitatively documented datasets under different future scenarios such as population, GDP, temperature, precipitation and so on [46]. Moreover, the combined multiple settings are more beneficial for modeling future trends than simulations based on single historical trajectories [47]. There has been an increasing number of studies using SSP-RCP scenarios to forecast future land-use changes [45,48,49,50,51,52].

As a key development area of the Yangtze Economic Belt of China, the middle reaches of the Yangtze River region (MRYRR) have superior natural conditions and various economic advantages. With the implementation of the “Development Plan of Urban Agglomeration in the Middle Reaches of the Yangtze River”, the regional land-use structure and spatial layout have undergone drastic changes due to rapid economic development [53], and cropland is being threatened by industrialization and urbanization. However, the three provinces located in the MRYRR are not only the main grain-producing areas of China due to their superior location, rich in water resources, photothermal conditions, and fertile soils, but also contain a large area of hills and mountains. Elucidating the past transformation of regional sloping cropland and predicting future trends are crucial to achieving the goals of cropland protection and grain security for the entire nation. However, how the areas of sloping cropland will change in the future and what the drivers of these changes have seldom been explored. Therefore, we selected the MRYRR as the study area for this work. We investigated the spatiotemporal distribution, changes, and slope spectrum of cropland with different slopes in the past and conducted future predictions for 2035, as this is the year that the development goals of the region should be completed according to the territorial spatial plans. We aimed to provide substantial guidance for decision-makers in rationally planning and utilizing cropland resources based on terrain features to prevent and control soil erosion while balancing ecological conservation, food security and sustainable urbanization under different scenarios.

## 2. Study Area

**Figure 1:**
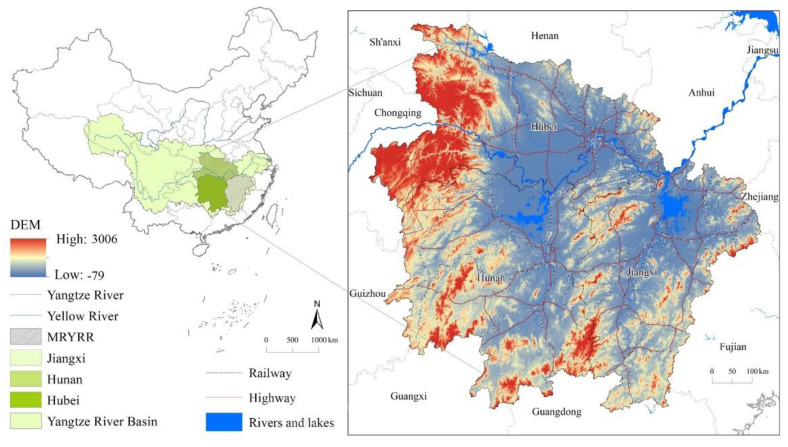
Location of the MRYRR.

The MRYRR is located in the center of China (108°21′–118°28′ E, 24°29′–33°20′ N) and encompasses Hubei, Hunan, and Jiangxi Provinces (Figure 1), with a total area of 56.47 × 10^4^ km^2^. There are diverse topographies, including plains, hills, and mountains, and the main urban agglomeration areas are located in the plains and hills. The mountain range in the area is inclined at an angle of 45°, with a “ω” shape, and the terrain is high in the southwest and low in the northeast. The land-use types in the region mainly include forestland, cropland, and built-up land, with areas of 32.65 × 10^4^ km^2^, 17.03 × 10^4^ km^2^ and 1.88 × 10^4^ km^2^, respectively, as of 2020. The economy in the area has developed rapidly in the past two decades, and the GDP reached 11.09 trillion yuan in 2020, a more than 11-fold increase as compared with the GDP of 0.9 trillion yuan in 2000. At the end of 2020, the urbanization rate was more than 60%.

## 3. Materials and Methods

### 3.1. Data Sources and Preconditioning

The original data used in this study included land-use, topography, and driving factor data. Land-use raster data of Hubei, Hunan, and Jiangxi provinces in 2000, 2005, 2010, 2015, and 2020, were derived from the Resource and Environment Science and Data Center of Chinese Academy of Sciences. The land-use types in this dataset are divided into six major categories (cropland, forestland, grassland, water area, built-up land, and unused land) and 25 sub-categories, with a spatial resolution of 30 m. The total accuracy is 94.3% [54], and the total accuracy of the 25 subcategories is 91.2% [53]. Topography data, namely digital elevation model data, were acquired from the ASTER GDEM v2 dataset, and the slope data had a spatial resolution of 30 m.

Land-use change is the result of interactions among natural, social and economic factors; therefore, we selected 14 factors with reference to relevant studies [41,48,55,56,57,58] based on the regional context of MRYRR. In terms of natural elements, factors including average annual temperature, average annual precipitation, soil type and habitat quality were selected. For socio-economic aspects, factors such as population, GDP, distance to highways, distance to trunk roads, distance to primary roads, distance to secondary roads, distance to tertiary roads, distance to railways, distance to rivers and distance to reservoirs were selected as driving factors. The data sources are listed in Table 1.

### 3.2. Methods

#### 3.2.1. PLUS-Based LUCC Simulation

The PLUS model integrates two sub-models, a rule-mining approach based on the Land Extension Analysis Strategy (LEAS) and a cellular automata model with a multi-type random patch seeds (CARS) mechanism. The model extracts changes in land-use types for two periods and explores the relationship between land-use changes and drivers using a random forest algorithm. Firstly, the growth probability of each land-use type in the study area is calculated by LEAS. Based on the selection of driving factors, the integrated LEAS module estimated the contribution of each driving factor to the change of each land type. Specifically, it analyzed the contribution values of each of the driving factors to the change of sloping cropland by using the random forest algorithm. Then, the number of pixels of different land-use types, the transformation matrix and the neighborhood weights of each land-use type were combined. Finally, future land-use change was simulated based on CARS. CARS with spatio-temporal dynamics (with temporal consistency) allows for new land-use patches to grow spontaneously, subject to the growth probability constraint.

To simulate the patch evolution of multiple land-use types, the easily operated PLUS model uses CARS mechanism based on threshold descent, which is implemented through the contribution calculation of driving factors overall probability. When the neighborhood effects of a land use type *k* are equal to 0, the growth probability surface ($P_{i,k}^{d=1}$) and overall probability ($OP_{i,k}^{d=1,t}$) of each land-use type can be obtained using a Monte Carlo approach as follows:

(1) $$OP_{i,k}^{d=1,t}=\{\begin{array}{c} P_{i,k}^{d=1}\times \left(r\times u_{k}\right)\times D_{k}^{t},\;if\Omega_{i,k}^{t}=0\;and\;r<P_{i,k}^{d=1}\\ P_{i,k}^{d=1}\times \Omega_{i,k}^{t}\times D_{k}^{t},\;all\;others \end{array}$$

where *r* is a random value ranging from 0 to 1; $u_{k}$ is the threshold for generating new land-use patches for land-use type *k*, which is determined by the model users; $\Omega_{i,k}^{t}$ is the proportion of land-use type *k* in the neighborhood of cell *i*; $D_{k}^{t}$ represents the gap between the quantity of land use and future demand for land use when the number of iterations is *t*. To control the generation of multiple land-use patches, a threshold descending rule for the competition process is proposed to limit both the organic and natural growth of all land-use types. If a new land-use type *c* wins the competition against land-use type *k*, a decreasing threshold *τ* is used to evaluate the candidate land-use type *c* by roulette wheel selection as follows:

(2) $$If\;\overset{N}{\underset{k=1}{\sum }}\left|G_{c}^{t-1}\right|-\overset{N}{\underset{k=1}{\sum }}\left|G_{c}^{t}\right|<Step\;Then,\;l=l+1\;\{\begin{array}{c} Change\;P_{i,c}^{d=1}>\tau \;and\;TM_{k,c}=1\\ No\;change\;P_{i,c}^{d=1}\leq \tau \;or\;TM_{k,c}=0 \end{array}\tau =\delta^{l}\times r1$$

where *Step* is the step size of the PLUS model, which approximates the land-use demand; *δ* is the decay factor of the decreasing threshold *τ*, with a value between 0 and 1, generally set according to expert experience; *r*1 is a normally distributed stochastic value ranging from 0 to 2 with a mean of 1; *l* is the number of decay steps. $TM_{k,c}$ is a transition matrix that determines whether land-use type *k* is allowed to convert to type *c*. Model parameter selection is based on the PLUS software tutorial.

#### 3.2.2. SSP-RCP-Based Scenario Simulation

We selected eight scenarios to predict future developments based on combining shared socioeconomic pathways (SSPs) and representation concentration pathways (RCPs). Each scenario represents a possible result of socioeconomic development and climate change. Scenario-based land-use forecasting can provide important information for assessing the long-term impacts and effectiveness of land-use management policies under different possible future conditions. The new scenarios in CMIP6 (Coupled Model Intercomparison Project Phase 6) are designed in conjunction with socio-economic and global climate change developments [46], and provide richer global climate model data for climate-change projections. In preparation for the IPCC’s 6th Assessment Report and as part of CMIP6, the Land-Use Harmonization (LUH2) project integrated five levels of SSPs and seven RCP targets into eight scenarios in 2100 and provided land-use projections for these scenarios: SSP1-1.9, SSP1-2.6, SSP2-4.5, SSP3-7.0, SSP4-3.4, SSP4-6.0, SSP5-3.4, and SSP5-8.5 [59]. However, the land-use simulation data provided by the LUH2 dataset may not match the characteristics of MRYRR in this study. Therefore, we referred to the study of Liao et al. [47] and modified the LUH2 dataset according to the characteristics of the study area. The adjusted LUH2 dataset used in the research was statistically corrected, and thus the demand for each land-use category under different scenarios of this study was obtained. Notably, the LUH2 project did not estimate changes in water area; therefore, in this study, we assumed that water area does not change under these scenarios and we did not simulate water area in land-use simulation models. A detailed description of each scenario is provided in Table 2.

The SSP-RCP-based scenario simulation was used to obtain the 2035 land-use map of the MRYRR by inputting the 2035 land-use quantities and the contribution of each of the driving factors into the CARS module of the PLUS model. The overall and provincial statistics of cropland and sloping cropland in MRYRR in 2000, 2005, 2010, 2015, 2020, and 2035 were conducted to obtain the changes of cropland and sloping cropland under different scenarios.

#### 3.2.3. Construction of a Cropland Slope Spectrum

We established a cropland slope spectrum to evaluate the spatial layout of sloping cropland quantitatively and visually. It is a graph with the cropland slope as the abscissa and the proportion of cropland area with a given slope in the total cropland area as the ordinate [60]. By accounting for the grid number and cropland grid number at each slope segment (with an interval of 1°) at regional and provincial scales, terrain slope spectrum curves and cropland slope spectrum curves for the actual land-use maps from 2000 to 2020 and the simulated land-use maps under the eight SSP-RCP scenarios in 2035 were plotted. When the proportion of cropland area in the total area of a slope segment is equal to that of the study area, the cropland slope spectrum curve and terrain slope spectrum curve cross. The change rate of the proportion of cropland area above this slope in the total cropland area in a period is termed “cropland climbing index” and the average change rate is termed “average cropland climbing index” (ACCI). When ACCI > 0, the proportion of cropland area above this slope in the total cropland area increases over time, i.e., cropland shows a climbing trend. The greater the ACCI, the stronger the increase.

## 4. Results

### 4.1. Simulation and Model Accuracy

**Figure 2:**
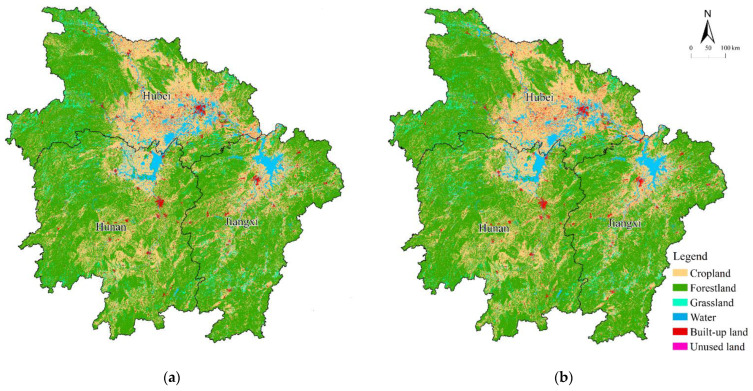
Real and simulated land-use maps of the MRYRR in 2010. (**a**) Real land use in the MRYRR according to official data in 2010. (**b**) Land use as simulated by the PLUS model based on data from 2005.

We simulated the land-use spatial pattern in 2010 using the PLUS model based on data from 2005 (Figure 2). The overall accuracy, Kappa coefficient, and Figure of Merit (FoM) were calculated to be 96.88%, 94.61%, and 0.15, respectively, which are the highest values when compared to other periods of time. This indicated that the PLUS model is well suited for simulating future land-use with high accuracy and confidence.

### 4.2. Variation Characteristics of Sloping Cropland during 2000–2020

#### 4.2.1. Distribution Characteristics of Sloping Cropland

**Figure 3:**
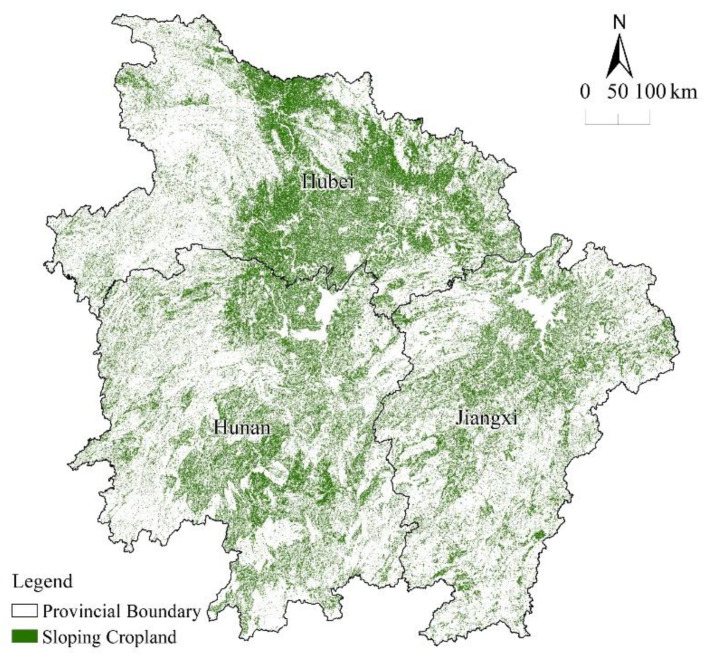
Distribution of sloping cropland in the MRYRR in 2020.

The spatial distribution characteristics of sloping cropland in the MRYRR were analyzed using the land-use data and slope map. Cropland with a slope above 2° is mainly concentrated in the central part of the study area. In Hubei Province, the sloping cropland is scattered in the east and center and distributed relatively densely as compared to that in the other two provinces. With a total sloping cropland area of 5.63 × 10^4^ km^2^, accounting for 81.93% of the province’s cropland, Hubei has the largest total sloping cultivated land area among the three provinces. In Hunan, the sloping cropland is mostly spread in the north and south, with a total area of 5.15 × 10^4^ km^2^, accounting for the highest proportion of 87.49%. The area of sloping cropland in Jiangxi is the smallest, with a total area of 3.67 × 10^4^ km^2^, accounting for 83.28% of the provincial cropland, and is distributed in the north and center of the province (Figure 3).

#### 4.2.2. Changing Characteristics of Sloping Cropland

**Figure 4:**
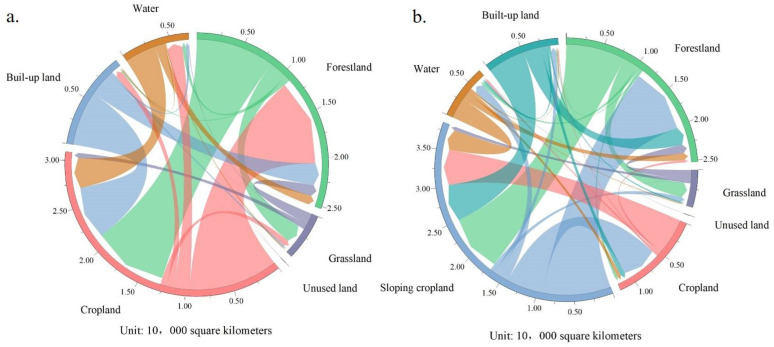
Land-use transfer flows in the MRYRR from 2000 to 2020. (**a**) Transfers between cropland and other land-use types. (**b**) Transfers between sloping cropland and other land-use types.

Between 2000 and 2020, the areas of cropland and sloping cropland both decreased overall, but the proportion of sloping cropland showed an upward trend. The total area of cropland decreased by 0.55 × 10^4^ km^2^ in the past 20 years, from 17.56 × 10^4^ km^2^ in 2000 to 17.01 × 10^4^ km^2^ in 2020, which is a 3.11% decrease. Cropland was mainly transferred to forests, built-up land, and water bodies, with areas of 0.79 × 10^4^ km^2^, 0.63 × 10^4^ km^2^, and 0.29 × 10^4^ km^2^, accounting for 43.68%, 34.83%, and 16.03%, respectively. Meanwhile, 0.87 × 10^4^ km^2^ of forests and 0.23 × 10^4^ km^2^ of water bodies were converted to cropland, accounting for 68.23% and 18.04% new cultivated land. The main transfer-in and -out types of sloping cropland were the same as those of cropland during the study period. In total, 2.16 × 10^4^ km^2^ of sloping cropland was transferred to other land-use forms: 0.78 × 10^4^ km^2^ to forests, 0.57 × 10^4^ km^2^ to built-up land, and 0.24 × 10^4^ km^2^ to water bodies. Meanwhile, 0.88 × 10^4^ km^2^ of forests and 0.18 × 10^4^ km^2^ of water bodies were transferred to sloping cropland, accounting for 62.7% of the total new sloping cropland (Figure 4).

### 4.3. Predictions of Sloping Cropland Based on Different Scenarios

**Figure 5:**
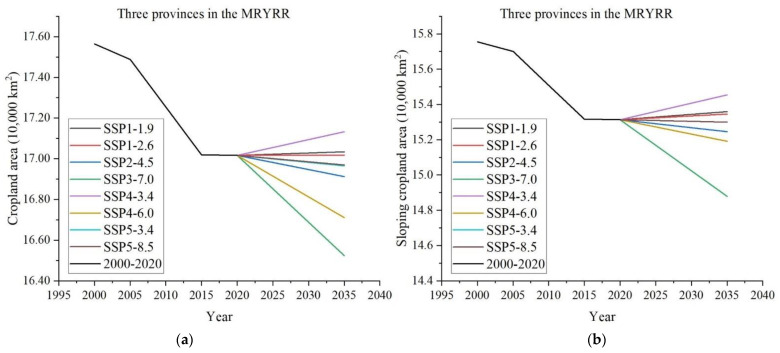
Predicted change trends in cropland and sloping cropland under different scenarios. (**a**) Predicted cropland areas in 2035. (**b**) Predicted sloping cropland areas in 2035.

Based on simulations in the PLUS model combined with SSP-RCP projections, changes in cropland and sloping cropland were predicted and showed the same trends under each scenario. The areas were estimated to either increase or decrease, but to quite different extents. Under the SSP4-3.4 scenario, the cropland area would maximally increase by 0.11 × 10^4^ km^2^, reaching a total area of 17.13 × 10^4^ km^2^ in 2035, whereas under the SSP3-7.0 scenario, it would sharply decrease by 0.50 × 10^4^ km^2^ to 16.52 × 10^4^ km^2^. The SSP1-2.6 scenario was predicted to be the most stable situation, in which cropland remained nearly the same as in 2020. Under the SSP2-4.5 and SSP4-6.0 scenarios, the cropland area would decrease by 0.12 × 10^4^ km^2^ and 0.3 × 10^4^ km^2^, respectively. As for sloping cropland, the largest area in 2035 was predicted to be 15.5 × 10^4^ km^2^ under the SSP4-3.4 scenario, whereas the smallest area of 14.88 × 10^4^ km^2^ was predicted under the SSP3-7.0 scenario. The amount of sloping cropland would decrease by 734 km^2^ under the SSP2-4.5 scenario (Figure 5).

### 4.4. Predictions of Cropland Slope Spectrum and ACCI

**Figure 6:**
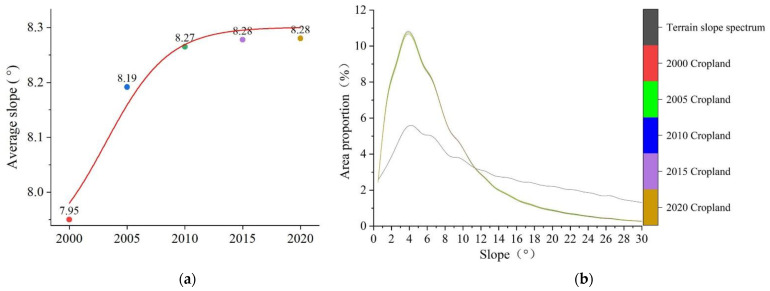
Average slopes and slope spectrum of cropland in the MRYRR in 2000–2020. (**a**) Changes in the average slopes of sloping cropland in 2000–2020. (**b**) Slope spectrum of cropland in 2000–2020.

In general, the cropland slope spectrum curves were in line with the terrain slope spectrum curve, with peaks between 4° and 5° (Figure 6b). The cropland slope spectrum curves, which showed a sharp increase and decrease, intersected with the terrain slope spectrum curve at around 11.5°, indicating that the proportion of sloping cropland with a slope lower than this level far exceeded the proportion of this terrain slope in the entire region.

From 2000 to 2020, the slope characteristics of cropland in the MRYRR changed obviously. In 2000, the average slope of cropland in the three provinces was 7.95°, and by 2020, the average slope reached 8.28°, with an increase of 0.33° (Figure 6a). The proportion of cropland below 2° gradually decreased, and the proportion of sloping cropland above 2° increased from 90.42% in 2000 to 90.60% in 2020. However, the proportion of cropland below 11.5° in the total area declined continuously during the twenty years, from 77.55% in 2000 to 76.68% in 2020, with the largest decline of 0.43% observed during 2005–2010, implying that the proportion of cropland above 11.5° had a slight increase.

**Figure 7:**
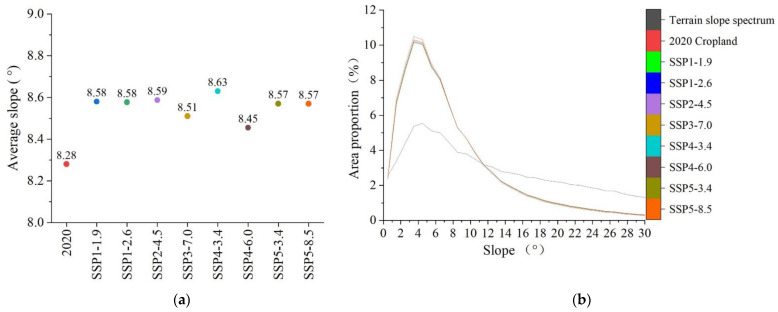
Average slopes and slope spectrum of cropland in the MRYRR in 2035 under different scenarios. (**a**) Predictions of the average slopes in 2035. (**b**) Slope spectrum of cropland in 2035.

By 2035, the average slope of cropland in the study area will further increase according to the simulations. The average cropland slope will maximally increase to 8.63° under the SSP4-3.4 scenario, an increase of 0.35° as compared with that in 2020. In contrast, the average slope of cropland will minimally increase to 8.45° under the SSP4-6.0 scenario, an increase of 0.17° as compared with that in 2020. The cropland slope spectrum and terrain slope spectrum curves will still cross around 11.5° in 2035; however, the proportion of sloping cropland below 11.5° will continuously decline to different extents. Under the SSP4-3.4 scenario, the proportion of clopped cropland will be 1.74% lower than in 2020. Under the SSP4-6.0 scenario, it will be only 0.87% lower than in 2020 (Figure 7).

According to the ACCI values for the different periods, the ACCI was higher than zero during 2000–2020, indicating that the cropland in the study area maintained a climbing trend. However, it is noteworthy that the intensity of the climbing trend showed up and down fluctuations between 2000 and 2020. The ACCI was the largest in 2005–2010, at 0.086%, indicating that the slope of cropland increased more significantly in this period than in the other periods. In 2010–2020, the ACCI decreased sharply to 0.0075%, indicating that the increase in sloping cropland was greatly slowing down.

**Figure 8:**
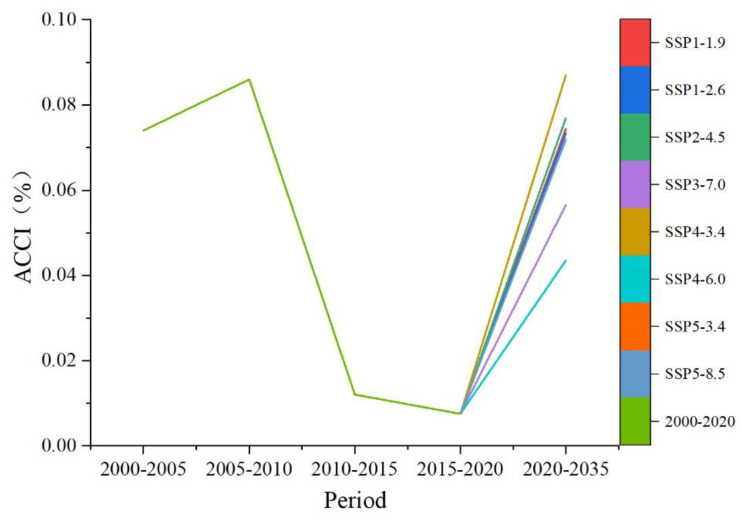
Changes in ACCI during 2000–2020 and 2020–2035.

By 2035, the ACCI is predicted to remain positive. Under the SSP4-3.4 scenario, it is projected to exceed that in 2005–2010 and reach 0.087%, and the intensity of cultivated land climbing will increase again. Under the SSP4-6.0 scenario, the ACCI is predicted to be the lowest, at 0.043%, which is still higher than in 2010–2020. Under the other scenarios, the ACCI is predicted to be in line with values seen in 2000–2005 (Figure 8).

### 4.5. Variations from the Perspective of Spatial Dimensions

**Figure 9:**
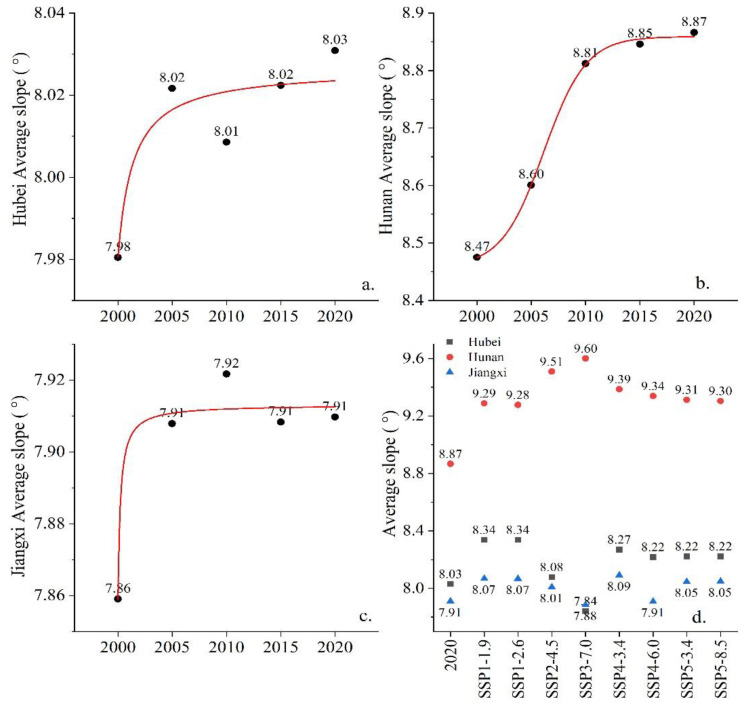
Changes of average slopes in the three provinces in 2000–2020 and predictions in 2035 under different scenarios. (**a**) Average slope in Hubei Province in 2000–2020. (**b**) Average slope in Hunan Province in 2000–2020. (**c**) Average slope in Jiangxi Province in 2000–2020. (**d**) Average slope in the three provinces in 2035 under different scenarios.

At the provincial level, the average slope of cropland in Hunan Province was the highest and changed the most in 2000–2020; from 8.47° in 2000 to 8.87° in 2020, an increase of 0.40°, which is higher than the average of the three provinces in the MRYRR. Until 2035, the average slope of cropland in Hunan Province will continue to increase. Among all future scenarios, the average slope was the largest at 9.6° under the SSP3-7.0 scenario and the lowest at 9.28° under the SSP1-2.6 scenario. For Jiangxi Province, the average slope of cropland was the lowest among the three MRYRR provinces, with little change between 2000 and 2020, a peak at 7.92° in 2010, and fluctuating around 7.91° in the following decade. By 2035, the average slope of cropland in Jiangxi Province will increase under most of the scenarios; it will reach the highest value of 8.09° under the SSP4-3.4 scenario. It will decrease to 7.89° only under the SSP3-7.0 scenario, while it will remain the same as in 2020 under the SSP4-6.0 scenario. For Hubei Province, the average slope of cropland fluctuated the least between 2000 and 2020. The average slope was 7.98° in 2000 and reached 8.03° in 2020. By 2035, the average slope of cropland in Hubei Province will increase, with the largest increase being 8.34° under the SSP1-1.9 and SSP1-2.6 scenarios, and it will decrease only under the SSP3-7.0 scenario, to 7.84° (Figure 9).

From 2000 to 2020, the peak value of the cropland slope spectrum in the three MRYRR provinces showed an overall downward trend, with Hunan Province having the largest decline of 0.3%, followed by Jiangxi Province (0.07%) and Hubei Province (0.06%). The decline of the proportion of cropland with lower slopes in total cropland indicated that the cropland has gradually moved to higher-slope areas. The change tendency of the sloping cropland proportion in the three provinces also revealed this feature. For the entire region, the proportion of sloping cropland in cultivated land in the three provinces increased from 90.42% in 2000 to 90.62% in 2010, and then slightly decreased in 2020 (90.60%). Similar change trends occurred in Hunan Province; the proportion of sloping cropland in cultivated land first increased to a maximum in 2010 (91.75%), with the fastest increase in 2005–2010, and then decreased to 91.64% in 2020. The proportion of sloping cropland in cultivated land in Jiangxi and Hubei Provinces maintained an upward trend. For Hubei Province, the proportion of sloping cropland in cultivated land was lower than the average of the three provinces; it rose from 89.35% in 2000 to 89.47% in 2010, and remained at the same level in 2010–2020. For Jiangxi Province, the proportion of sloping cropland in cultivated land was higher than the average of the three provinces; it increased from 90.78% in 2000 to 90.87% in 2020, with the fastest increase in 2000–2005.

#### 4.5.1. Sloping Cropland Changes and Trends in Hubei Province

**Figure 10:**
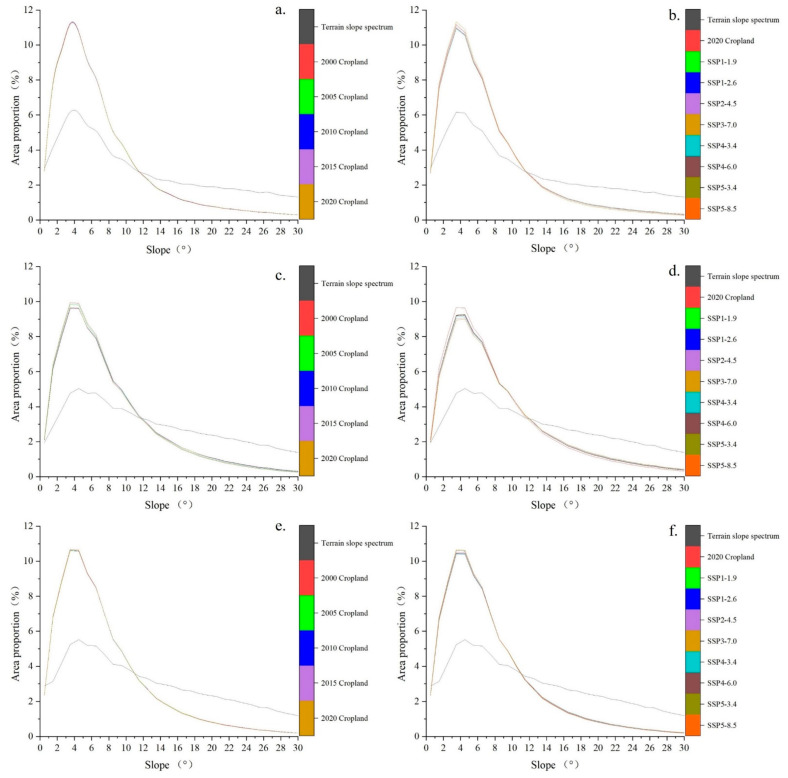
Cropland slope spectra for Hubei, Hunan, and Jiangxi during 2000–2020 and predictions of 2035 under different scenarios. (**a**) Cropland slope spectra for Hubei during 2000–2020; (**b**) Cropland slope spectrum simulations for Hubei in 2035; (**c**) Cropland slope spectra for Hunan during 2000–2020; (**d**) Cropland slope spectra simulations for Hunan in 2035; (**e**) Cropland slope spectra for Jiangxi during 2000–2020; (**f**) Cropland slope spectra simulations for Jiangxi in 2035.

As for the entire region, for Hubei Province, the slope spectrum curves of cultivated land and the terrain slope spectrum curve crossed at around 11.5° in 2000–2020 and 2020–2035 (Figure 10a,b). The proportion of sloping cropland above 11.5° in the total cropland area showed an overall upward trend; it increased from 20.97% in 2000 to 21.27% in 2020. This indicates that cultivated land tends to move to high-slope areas in Hubei Province.

**Figure 11:**
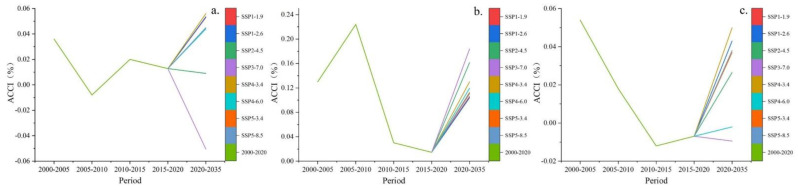
ACCI for Hubei (**a**), Hunan (**b**), and Jiangxi (**c**) Province, 2000–2035.

According to the ACCI values for Hubei Province in the different periods, the average slope of cultivated land was fluctuating during 2000–2020, with the highest in 2000–2005 and the lowest in 2005–2010. By 2035, the ACCI of Hubei Province will be the largest under the SSP4-3.4 scenario and exceed that seen in 2000–2005. Under the SSP2-4.5 scenario, the cultivated land in Hubei Province will maintain a climbing development (in line with the current development trend), but the climbing speed will slow down. Under the SSP3-7.0 scenario, the ACCI of Hubei Province will be the smallest, below zero, indicating that the cultivated land will develop towards low-slope land (Figure 11a).

#### 4.5.2. Sloping Cropland Changes and Trends in Hunan Province

For Hunan Province, the slope spectrum curves of cultivated land and the terrain slope spectrum curve cross around 12° in 2000–2020 and 2020–2035 (Figure 10c,d). The proportion of cultivated land above 12° in total cultivated land shows an overall upward trend, with proportions of 21.55%, 22.20%, 23.32%, 23.47%, and 23.56% in 2000, 2005, 2010, 2015, and 2020, respectively, indicating that the cultivated land in Hunan Province tend to move to higher-slope areas.

The ACCI variation trend for Hunan Province was the same as that for the three provinces in the MRYRR. The ACCI was consistently above zero in 2000–2020 and 2020–2035, indicating that the cultivated land in Hunan Province will continue to climb to higher-slope areas; however, the intensity of cultivated land climbing fluctuated. In 2005–2010, the ACCI was the highest, at 0.22%, which was higher than the average of the three provinces, and cultivated land climbing was the most intense. In 2010–2020, the ACCI decreased sharply to 0.03% in the first five years and then continued to decline to 0.015% in 2020 as cultivated land climbing slowed down. By 2035, the ACCI of Hunan Province is expected to increase, and cultivated land climbing will continue to accelerate. The ACCI will be the highest under the SSP3-7.0 scenario, reaching 0.18%, and will reach the lowest values under the SSP1-2.6 scenario, at 0.10%; however, the climbing intensity is much higher than in 2010–2020 (Figure 11b).

#### 4.5.3. Sloping Cropland Changes and Trends in Jiangxi Province

For Jiangxi Province, the slope spectrum curves of cultivated land and the terrain slope spectrum cross around 11° in 2000–2020 and 2020–2035 (Figure 10e,f). The proportion of cultivated land above 11° in total cultivated land first increased and then decreased; it reached a maximum value of 21.74% in 2010 and decreased to 21.65% in 2020. Overall, cultivated land in Jiangxi Province tends to move to higher-slope areas.

The ACCI in Jiangxi Province was the highest in 2000–2005, which implies that the cropland climbing was the most intense in this period. In 2005–2010, the speed of cultivated land climbing slowed down. In 2010–2020, the ACCI was below zero, with fluctuations, indicating that the average slope of cultivated land was decreasing. By 2035, the ACCI of Jiangxi Province will be the largest under the SSP4-3.4 scenario, at 0.05%, which is comparable to the level in 2000–2005. If the current development trend is maintained (SSP2-4.5), the cultivated land in Jiangxi Province will start to climb again, but at a lower speed than in 2000–2005. Under the SSP3-7.0 and SSP4-6.0 scenarios, the ACCI of Jiangxi Province will be below zero, and the cultivated land will move towards low-slope areas at different speeds (Figure 11c).

### 4.6. Drivers of Sloping Cropland Change

**Figure 12:**
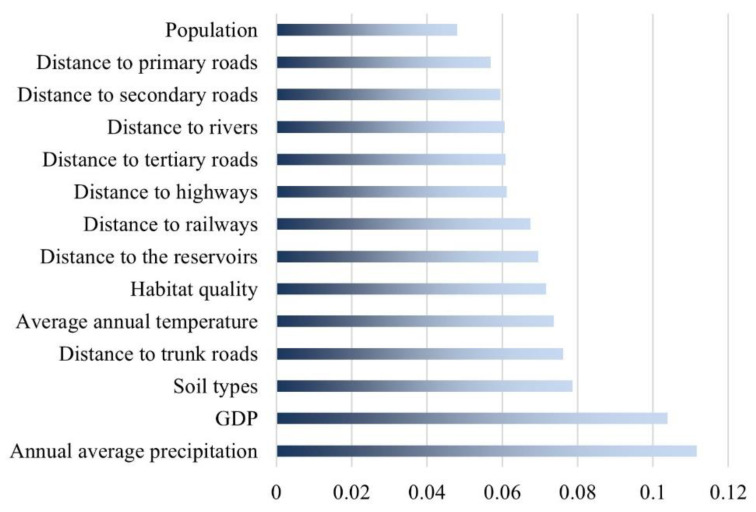
Contributions of factors to the growth of sloping cropland area.

**Figure 13:**
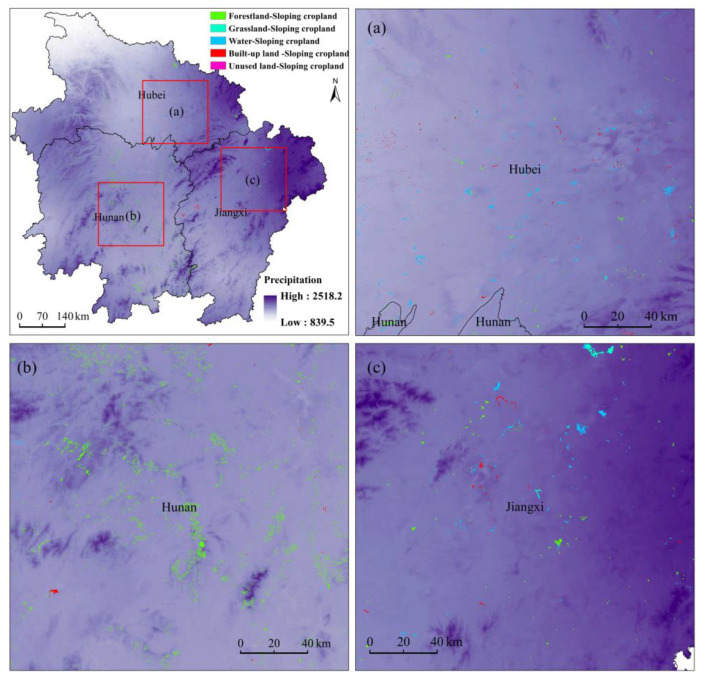
Spatial relationships between annual average precipitation and new sloping cropland in 2005–2010. (**a**) Selected typical region in Hubei Province. (**b**) Selected typical region in Hunan Province. (**c**) Selected typical region in Jiangxi Province.

The graph below shows a ranking of the 14 factors driving sloping cropland expansion selected in this study based on their contribution (Figure 12). The main drivers of sloping cropland expansion were average annual precipitation and GDP, of which average annual precipitation was the most influential factor. The new sloping cropland was concentrated in the southern (Hunan) and eastern regions (Jiangxi), with relatively high annual average precipitation. There was also limited new sloping cropland in the northern region (Hubei), albeit being fragmented and very scattered. In Hubei Province, the severely fragmentated new sloping cropland was mostly converted from built-up land, whereas the sloping cropland converted from forestland or water areas mainly showed a striped or group distribution. In Jiangxi Province, the sloping cropland converted from built-up land was often distributed in groups or strips, and it was more concentrated than in Hubei Province. The sloping cropland converted from water areas and grassland mostly showed a cluster distribution, whereas the sloping cropland converted from forestland mostly showed a dot configuration, with very scattered distribution. In Hunan Province, the new sloping cropland was more agglomerated in clusters than in other provinces. The sloping cropland converted from water areas and forestland was mostly clustered in groups or blocks, whereas that converted from built-up land was more scattered (Figure 13).

**Figure 14:**
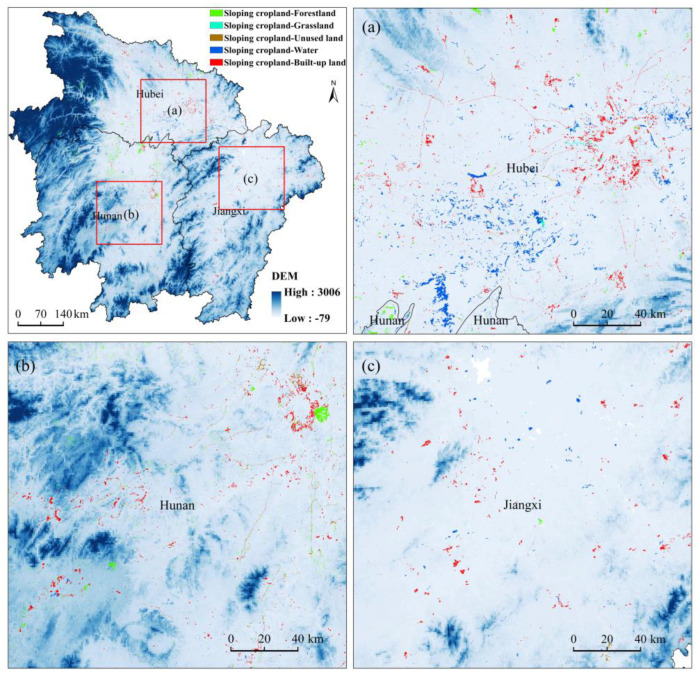
Spatial distribution of sloping cropland transfers to other land uses. (**a**) Selected typical region in Hubei Province. (**b**) Selected typical region in Hunan Province. (**c**) Selected typical region in Jiangxi Province.

During the sloping cropland transition-out from 2005 to 2010, cropland was mostly transferred to forestland, built-up land, and water areas. By overlaying the elevation map and the area of sloping cropland transition-out (data not shown), we found that in the plain area with a low altitude, due to the intense human activities, cropland was more disturbed and a large amount of cultivated land with a scattered distribution was transferred to built-up land and water areas, whereas in the mountainous areas with low human activity, cropland was transformed into forestland, with a strip or group distribution (Figure 14).

## 5. Discussion

Rational utilization of sloping cropland is of great significance to both ensure food security and achieve sustainable environmental management. The simulation results predict changing trends in sloping cropland under different developmental scenarios in the future and can provide relevant decision-making suggestions for regional cultivated land policy formulation. The predicted areas of cropland and sloping cropland are different under the eight scenarios in 2035, while the orders of the amounts of cropland and sloping cropland are the same. SSP4-3.4 has the largest predicted areas, followed by SSP1-1.9, SSP1-2.6, SSP5-8.5, SSP5-3.4, SSP2-4.5 and SSP4-6.0, and SSP3-7.0 has the smallest predicted areas. Predictions under the SSP4-3.4, SSP1-1.9 and SSP1-2.6 show an increase with different degrees of both cropland and sloping cropland in 2035 compared to 2020, while scenarios of SSP3-7.0, SSP4-6.0 and SSP2-4.5, respectively, indicate an obvious declining trend. The scenario of SSP4-3.4 has relatively strict climate policies, which will lead to higher carbon prices. A large amount of land will be used for bioenergy production, which will lead to a large increase in cropland and sloping cropland. Compared with the SSP4-3.4, scenarios under SSP1-1.9 and SSP1-2.6 with the goals of limiting environmental impact on biodiversity loss and increasing the usage of bioenergy, respectively, thus lead to a slow increase in cropland and sloping cropland. The SSP5-8.5 and SSP5-3.4 scenarios are dominated by economic development, with rapid growth in food and feed demand. The SSP2-4.5 scenario, representing business-as-usual development, is a low-stability scenario, which may cause the slow decline of cropland and sloping cropland when the nation aims to achieve sustainable development. However, progress may be slow. As a result, the areas of cropland and sloping cropland will continue to decline in the same trend from 2020–2035 as they did from 2000–2020. The SSP4-6.0 scenario encourages afforestation in high- and middle-income areas with strong environmental policies, where cropland and sloping cropland are occupied by forest, resulting in a rapid decline in the area of cropland. The sharp decrease trend of predicted areas of cropland and sloping cropland under the SSP3-7.0 scenario may be partly attributed to urban expansion and partly to mitigation of the global warming effect with a steady increase of emissions and temperatures. It is worth mentioning that the decline of sloping cropland in SSP4-6.0 is significantly smaller than that of cropland, mainly because most of the high- and middle-income areas with strong environmental policies are plains, and the conversion of sloping cropland to forestland is less than that of cropland.

Based on the comparative analysis above, we found that development scenarios with relatively strict climate policies and competitive mechanisms can protect cropland much better than extreme ecological protection and urban development scenarios. Therefore, in the future development of the MRYRR, attention should be paid to controlling the occupation of cropland by urban expansion to prevent its rapid decline in various areas, which may threaten food security. In addition, under the SSP5-8.5 scenario in which the area of cropland decreases and the proportion of sloping cropland gradually increases, local governments should make more rational use of cropland resources. In the MRYRR, cropland below 15° constitutes the main body of cultivated land resources, generally accounting for more than 85% of the total area. Moreover, cropland below 15° is mainly used for high-yield grain production, which is an important guarantee for maintaining agricultural production. At slopes of 15–25°, the quality of cropland generally declines because soil and water erosion is more serious than lower slopes. Therefore, when using cropland with slopes of 15–25°, water-saving measures, including biological and engineering measures, should be adopted. Especially, slope–terrace transformation projects should be implemented to improve the sustainable production capacity of sloping cropland. Cultivated land with slopes above 25° is generally not suitable for farming and is best returned to forest or grassland.

As the sloping cropland is the main source of soil erosion which can lead to global soil degradation [19], the predicted increase of average slope of sloping cropland in the study area will result in huge pressure on soil and water conservation under the SSP-RCP scenarios. The MRYRR includes the Three Gorges Reservoir Area and Danjiangkou Reservoir Area which are the key soil erosion areas. Many risks, such as landslides and mudslides, may occur if too much cropland were cultivated in high-slope areas. Precipitation is one of the main driving factors of soil erosion, and rainfall erosivity is used as basic indicator to assess soil erosion potential and ecological sensitivity [61]. In this study, the rainfall was estimated to be the most contributing factor to the growth of sloping cropland within the region. Under the combined effect of water and slope, this trend indicates that there will be an increasing risk of rainfall erosivity in the basin and soil water erosion [62], which can be very unfavorable to the prevention and control of soil water loss for not only the study area but also the entire Yangtze River Basin. A study in the karst region of southern China, including Hubei and Hunan of MRYRR, demonstrates the very critical role of the magnitude of rainfall erosivity in soil and water conservation in the fragile ecological environment [63]. Therefore, prevention of cultivating sloping land from forests and the early warning of sloping cropland in the heavy rainfall season can be extremely important.

Among the contributing factors of sloping cropland change, natural environmental parameters mostly determine the overall spatial layout of cropland, while socio-economic factors largely propel the evolution direction and conversion quantity. In this study, the increase of sloping cropland in the MRYRR was most driven by two factors: the average annual precipitation and GDP. Precipitation can bring about more nutrient-rich soil which plays a significant role in grain production [64], and hydrothermal conditions are becoming widely suitable for cultivation as the climate gets warmer. Additionally, the arable land-balancing policy, lasting for over twenty years, has made local governments habituated to supplementing cropland in remote rural areas. The more developed the economy is, the more cropland it can replenish. Spatially, as shown in Figure 14, sloping cropland in plain areas, where cities and towns are extensively distributed, was very likely to be converted to built-up land. Furthermore, the variations between spatial change configuration of sloping cropland in different regions can be easily observed. In plains, at lower altitudes, because of the severe fragmentation of land-use patches due to human activities, a large amount of sloping cropland is transferred to built-up land in a scattered configuration, whereas its distribution is relatively concentrated in areas surrounding cities. In the mountains, which are less-affected by human activities, cropland is mostly transferred to forestland in strips or groups.

This study had some limitations. Firstly, the simulation of sloping cropland was integrated in the PLUS-based land-use simulations. Further studies are needed to specifically evaluate the changes in cropland, as the driving factors vary according to development situations. Secondly, previous research has shown that paddy farming in water-rich regions can mitigate soil erosion and gain more economic benefits [65]. This study, however, did not consider the types of agricultural production, such as paddy land or dry land, indicating that the prediction has a further refinement. Thirdly, the research scale can be extended from the provincial level to the municipal or even county level, to better compare and study the slope spectrum changes of cropland at different scales and analyze the intensity of cropland climbing.

## 6. Conclusions

In this study, we simulated the spatiotemporal changes in sloping cropland in the MRYRR under eight SSP-RCP scenarios and analyzed the contribution of different factors to the change in sloping cropland. The following key conclusions were drawn:

(1) The areas of cropland and sloping cropland in the MRYRR both showed an overall downward trend in 2000–2020, whereas the proportion of sloping cropland presented an overall upward trend. It is expected that by 2035, the cropland area will exhibit various changing trends under different scenarios. The increase will be largest under the SSP4-3.4 scenario, while the decrease will be largest under the SSP3-7.0 projection. If the current trend continues (SSP2-4.5), the area of cropland will continue to decline.
(2) The average slope of cultivated land in the MRYRR gradually increased by 0.33° in 2000–2020 and is expected to further increase until 2035. The highest average slope of cropland occurred under the SSP4-3.4 scenario and the lowest occurred under the SSP4-6.0 scenario. At the provincial level, in 2035, the average slope of cultivated land will be the highest in Hunan Province and the lowest in Jiangxi Province, with a moderate value for Hubei Province.
(3) According to the ACCI values in different periods, the cropland in the MRYRR will maintain a climbing development in 2020–2035. The intensity of cropland climbing will be highest under the SSP4-3.4 scenario both for the entire region and the three provinces, exceeding that in 2005–2010.
(4) Among the 14 drivers selected, the average annual precipitation and GDP contributed the most to the expansion of sloping cropland. Under the trend of climate warming and the control of the arable land balancing policy, rich rainfall and a high level of economic development will drive the cropland in plains to the slopes. The flow-out of sloping cropland is greatly affected by human activities. The findings of this study can provide new insights with strong data support for decision-making in the rational utilization of cultivated land, in addition to soil and water conservation.

## Figures and Tables

**Table 1:** Description of the data used for future land-use simulations.

Data Type	Data	Spatial Resolution	Data Resource
Land-use data	Land-use data of Hubei, Hunan, and Jiangxi Provinces in 2000, 2005, 2010, 2015, and 2020	30 m	Resource and Environment Science and Data Center of Chinese Academy of Sciences [online]. Available from: http://www.resdc.cn/Datalist1.aspx?FieldTyepID=1,3 (Accessed: 20 December 2021)
Environmental data	Soil type	1 km	National Tibetan Plateau Data Center [online]. Available from: https://data.tpdc.ac.cn/zh-hans/data/611f7d50-b419-4d14-b4dd-4a944b141175/ (Accessed: 15 December 2021)
	Habitat quality		See reference [1].
	Average annual temperature	0.5°	WorldClim 2.1 [online]. Available from: https://www.worldclim.org/data/index.html (Accessed: 17 December 2021)
	Average annual precipitation		
Socio-economic data	GDP	1 km	Resource and Environment Science and Data Center of Chinese Academy of Sciences [online]. Available from: https://www.resdc.cn/data.aspx?DATAID=354 (Accessed: 17 December 2021)
	Population		
	Roads and railways	Vector	OpenStreetMap [online]. Available from: https://www.openstreetmap.org/#map=5/38.007/-95.844 (Accessed: 18 December 2021)
	Reservoirs and rivers	Vector	National Catalogue Service for Geographic Information [online]. Available from: https://www.webmap.cn/main.do?method=index (Accessed: 20 December 2021)

**Table 2:** Description of SSP-RCP scenarios.

Scenario	Description
SSP1-1.9	The SSP1 scheme (sustainable path) is a sustainable development scheme emphasizing eco-friendly development. SSP1-1.9 describes an environmentally conscious world with the goal of limiting the environmental impact on biodiversity loss and food consumption.
SSP1-2.6	From the perspective of land use, an important policy under this scenario is to increase bioenergy use and to combine carbon-capture and storage technologies to avoid deforestation policies to reduce indiscriminate logging. “Ecological protection” scenario.
SSP2-4.5	SSP2 represents an intermediate path, with global and national institutions committed to achieving the sustainable development goals under the scheme; however, progress has been slow. SSP2-4.5 is a low-stability scenario that represents a scenario for sustaining current socio-economic, scientific, and technological development trends. “Development as usual” scenario.
SSP3-7.0	Each country under the SSP3 scheme (regional competitive path) strives to develop on the basis of achieving energy and food-security goals. The SSP3-7.0 scenario has a radiated forcing levels close to 7.0 W/m^2^, resulting in a significant expansion of crop land and pasture land around the world, leading to large-scale deforestation.
SSP4-3.4	SSP4 represents an unbalanced path, with more strict climate policies, which will lead to higher carbon prices. The increase in carbon prices will have a significant impact on energy and land use, with the use of a large amount of land for the production of biological energy, which will lead to a substantial increase in cropland.
SSP4-6.0	SSP4-6.0 adopts a mild climate policy in which global cropland and pastures expand moderately, and a mild climate policy encourages afforestation in high- and middle-income areas with strong environmental policies.
SSP5-3.4	SSP5 represents a development path for high fossil-fuel consumption. Under the SSP5-RCP3.4 scenario, there will be more cropland expansion around the world due to the large-scale deployment of second-generation bioenergy crops after 2040.
SSP5-8.5	Global fossil fuel is overused under the SSP5-RCP8.5 scenario, with global food demand doubling in this century and greenhouse gas emissions tripling. Due to the rapid growth of food and feed demand, cropland extensively expands into pasture and woodland. “Economic growth” scenario.

## Data Availability

Publicly available datasets were analyzed in this study. This data can be found here: Resource and Environment Science and Data Center of Chinese Academy of Sciences [online]. Available from: http://www.resdc.cn/Datalist1.aspx?FieldTyepID=1,3 (Accessed: 20 December 2021); National Tibetan Plateau Data Center [online]. Available from: https://data.tpdc.ac.cn/zh-hans/data/611f7d50-b419-4d14-b4dd-4a944b141175/ (Accessed: 15 December 2021); WorldClim [online]. Available from: https://www.worldclim.org/data/index.html (Accessed: 17 December 2021); Resource and Environment Science and Data Center of Chinese Academy of Sciences [online]. Available from: https://www.resdc.cn/data.aspx?DATAID=354 (Accessed: 17 December 2021); OpenStreetMap [online]. Available from: https://www.openstreetmap.org/#map=5/38.007/-95.844 (Accessed: 18 December 2021); National Catalogue Service for Geographic Information [online]. Available from: https://www.webmap.cn/main.do?method=index (Accessed: 20 December 2021).
